# Glioblastoma research on zebrafish xenograft models: a systematic review

**DOI:** 10.1007/s12094-023-03258-7

**Published:** 2023-07-03

**Authors:** Alexandra Pliakopanou, Ilias Antonopoulos, Nikolia Darzenta, Iliana Serifi, Yannis Vasilios Simos, Andreas Panagiotis Katsenos, Stefanos Bellos, George Athanasios Alexiou, Athanasios Petros Kyritsis, Ioannis Leonardos, Patra Vezyraki, Dimitrios Peschos, Konstantinos Ioannis Tsamis

**Affiliations:** 1 Laboratory of Physiology, Faculty of Medicine, School of Health Sciences, University of Ioannina, 45110 Ioannina, Greece; 2 Laboratory of Biological Chemistry, Faculty of Medicine, School of Health Sciences, University of Ioannina, 45110 Ioannina, Greece; 3 Neurosurgical Institute, University of Ioannina, 45500 Ioannina, Greece; 4 Zoology Laboratory, Department of Biological Application and Technology, University of Ioannina, 45110 Ioannina, Greece

**Keywords:** Zebrafish, Glioblastoma, Xenograft, Tumor model, Preclinical model

## Abstract

Glioblastoma (GBM) constitutes the most common primary brain tumor in adults. The challenges in GBM therapeutics have shed light on zebrafish used as a promising animal model for preclinical GBM xenograft studies without a standardized methodology. This systematic review aims to summarize the advances in zebrafish GBM xenografting, compare research protocols to pinpoint advantages and underlying limitations, and designate the predominant xenografting parameters. Based on the PRISMA checklist, we systematically searched PubMed, Scopus, and ZFIN using the keywords “glioblastoma,” “xenotransplantation,” and “zebrafish” for papers published from 2005 to 2022, available in English. 46 articles meeting the review criteria were examined for the zebrafish strain, cancer cell line, cell labeling technique, injected cell number, time and site of injection, and maintenance temperature. Our review designated that AB wild-type zebrafish, Casper transparent mutants, transgenic Tg(fli1:EGFP), or crossbreeding of these predominate among the zebrafish strains. Orthotopic transplantation is more commonly employed. A number of 50–100 cells injected at 48 h post-fertilization in high density and low infusion volume is considered as an effective xenografting approach. U87 cells are used for GBM angiogenesis studies, U251 for GBM proliferation studies, and patient-derived xenograft (PDX) to achieve clinical relevance. Gradual acclimatization to 32–33 °C can partly address the temperature differential between the zebrafish and the GBM cells. Zebrafish xenograft models constitute valuable tools for preclinical studies with clinical relevance regarding PDX. The GBM xenografting research requires modification based on the objective of each research team. Automation and further optimization of the protocol parameters could scale up the anticancer drug trials.

## Introduction

Glioma is the most common malignant form of the central nervous system (CNS) neoplasms and derives from the glial cells that surround and support neurons in the brain, including astrocytes (i.e., astrocytomas), oligodendrocytes (i.e., oligodendrogliomas), and ependymal cells (i.e., ependymomas) [1]. Gliomas have been classified into clinical grades of ascending malignancy based on histology and immunochemistry by the WHO [2]. Grade 4 astrocytoma, namely glioblastoma (GBM), is the most malignant and aggressive primary brain tumor displaying the worst prognosis with less than 5% of the patients surviving 5 years following diagnosis. [3] GBMs invade the nearby brain tissue but generally do not spread to distant organs.

GBM cells display challenges, constituting a highly heterogeneous population with unique mutational profiles and dissimilar phenotypes in terms of morphology, self-renewal, proliferative capacity, and therapeutic sensitivity. In addition, GBM cellular plasticity [4] promotes a dedifferentiated CD133^+^ stem-like cell population reported as an unavoidable contributor to therapy evasion [5].

Despite the progress made, treatment of GBM remains a complex and difficult challenge. The standard therapeutic approach to GBM includes surgical resection, gross total, or subtotal, depending on the morphology, localization, and vascularity of the tumor [5–7]. Resection is followed by radiotherapy, whereas GBM tumors display radioresistance due to upregulated repair machinery. Concomitant and adjuvant chemotherapy with temozolomide (TMZ) induces tumor cell death. However, TMZ can harm healthy cells and GBM tumor cells exhibit reduced sensitivity to TMZ when a DNA repair gene (*MGMT*) is overexpressed [8]. These limitations as well as the moderate effectiveness of other approved drugs targeting GBM call for novel treatment strategies.

Existing drug repurposing has emerged as an attractive strategy since the development of new therapeutic approaches can be high cost and slow paced. At the same time, novel treatment strategies, such as tumor treating fields and laser interstitial thermal therapy, are being investigated and display encouraging results [9]. Despite the low tumor mutational burden and the immunosuppressive environment of GBM, immunotherapeutic strategies have been explored including checkpoint blockade to suspend the T-cell downregulatory mechanism, engineering-enhanced chimeric antigen receptor T cells (CAR-T cells) therapy, vaccine-based strategies, and oncolytic viruses [9–11].

The multifactorial, multistep nature of carcinogenesis resulting from complex interactions of cancer cells with their microenvironment and the whole organism calls for in vitro cancer models interpreting the molecular mechanisms of tumor progression, complemented by in vivo models, deciphering the multicellular interactions of tumor progression [12]. Animals are necessarily used for preclinical brain tumor research including chemically induced, genetically engineered, and xenograft animal models, with the latter displaying high clinical relevance [5, 13, 14]. However, traditionally used mammalian models (e.g., rodents) come with limitations, such as high cost, time consuming, and ethically questionable operation, rendering them inappropriate for large-scale anticancer drug screening. Recently, zebrafish (*Danio rerio*) have emerged as a promising alternative for in vivo studies, allowing for translatable brain cancer research and high-throughput drug screening. Their cost-efficient husbandry, high fecundity and rapid development *ex utero*, the small size and transparency of their embryos, as well as the availability of well-characterized zebrafish strains with fully sequenced genome—showcasing high genetic similarity to humans (70% genetic homology), and, thus, conferring interspecies biological processes conservation—configure zebrafish as a valuable tool to recapitulate glioblastoma in vivo with minimally invasive real-time imaging techniques at single-cell resolution [5, 12, 15].

It is apparent that successful bench-to-bedside translation of glioblastoma research findings into therapeutic interventions depends on the selection of proper experimental animal models. The current paper aims to summarize recent advances in using zebrafish as a model in cancer studies with specific focus on glioblastoma, collate zebrafish xenograft models of different developmental stage and xenograft injection site to pinpoint advantages and underlying limitations, and discuss future challenges in zebrafish xenotransplantation.

## Methods

**Fig. 1:**
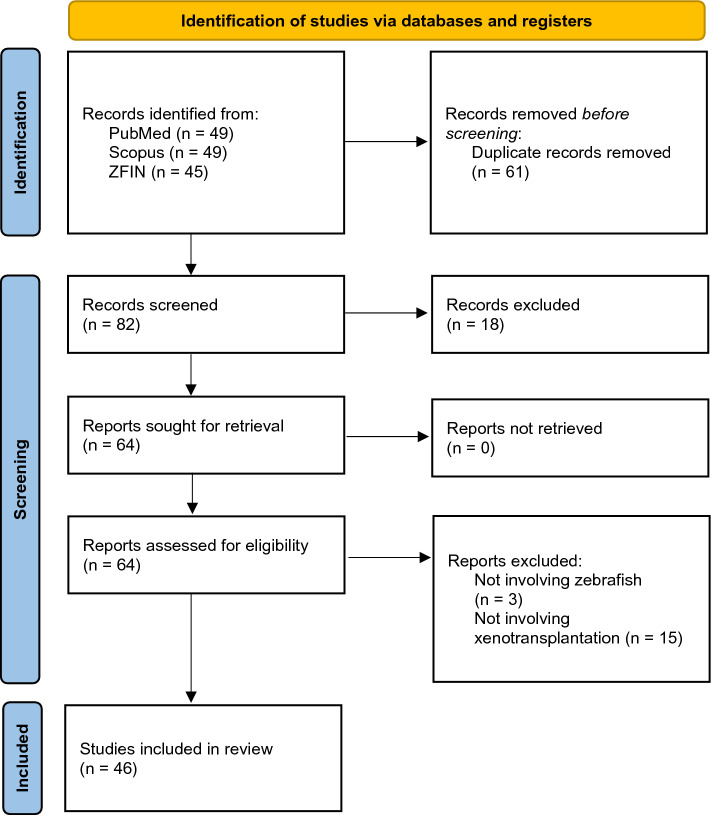
PRISMA flow diagram demonstrating the search strategy, the number of records identified, and the excluded/included papers throughout the screening process

This systematic review was conducted in line with the Preferred Reporting Items for Systematic Reviews and Meta-Analyses (PRISMA) statement [16] to report reliably structured information (Fig. 1).

### Eligibility criteria

We considered original studies using glioma cell lines and patient-derived xenotransplants in zebrafish and excluded studies in neuroblastoma embryonal tumor xenograft models.

### Information sources and search strategy

We systematically searched PubMed, Scopus, and ZFIN (Zebrafish Information Network) using the concepts “glioblastoma,” “xenotransplantation,” and “zebrafish” as keywords for the search syntax. Searches were restricted to texts available in English and published from 2005 to 2022 (October 30).

### Selection process

143 records were identified in total (PubMed *n* = 49, Scopus *n* = 49, ZFIN *n* = 45). Duplicate records were removed, and single records were screened by two of the authors independently. Review articles and book chapters were excluded. Title and abstract reviewing excluded irrelevant studies and the full manuscript was evaluated in uncertain cases to ensure compliance with the eligibility criteria. Studies involving neoplasms apart from higher-grade gliomas/glioblastoma and/or employing genetic manipulation (e.g., gene orthologs, gene knockdown with antisense morpholino oligonucleotides) without xenotransplantation or using solely non-xenografted zebrafish as toxicity screening in vivo model were eliminated. We solved any sorting discordances by consensus and cross-checking between the authors was employed to guarantee proper final article selection for the review.

### Data collection process

The articles meeting the review criteria were inserted into a table to facilitate the classification, comparison, and analysis of the findings (Table 1).

**Table 1:** Zebrafish GBM xenografting protocol parameters extracted from the reviewed articles, including the zebrafish strain, the cancer cell line, the cell labeling technique, the injected cell number, the time and site of injection, the xenograft maintenance temperature, and their corresponding protocol reference (if any)

Reference	Zebrafish strain	Cancer cell line	Cell labelling	Cell number (Suspension volume, Concentration)	Time of injection	Injection site	Xenograft maintenance temperature	Based on
Ai et al. [52]	Tg(kdrl:EGFP) × Nacre	U251-MG, U87-MG, patient-derived (#109, #24, GSCs BNI-21, BNI-23)	mCherry, CFSE fluorescent dye	100 (3–5 nL)	72 hpf	Orthtopic; midbrain (optic tectum)	33 °C	Pudelko (2018)
Almstedt et al. [34]	Casper (roy;nacre), Tg(kdrl:mCherry)	Patient-derived	GFP, luciferase	150	24 hpf	Orthtopic; midbrain	33 °C	
Barbieri et al. [24]	Wild type; AB	GSCs (GBM3)	ZsGreen	150–200	48 hpf	Orthtopic; hindbrain	32 °C	Grissenberger (2021)
Gabler et al. [39]	mitfa^b692/b692^; ednrba^b140/b140^	BTL1528, FGFR4-KD	GFP	Not mentioned	48 hpf	Heterotopic; yolk sac	34 °C	Martinez-Lopez (2021)
Liang et al. [35]	Casper (roy;nacre)	GSCs (GSC23)	CellTracker™ Green CMFDA	250	24 hpf	Heterotopic; perivitelline space (PVS)	34 °C	
Lubanska et al. [58]	Not mentioned	U251-MG, patient-derived	Fluorescently labelled	(9.2 nL, 10^6^cells/mL)	72 hpf	Heterotopic; yolk sac (centre)	Not mentioned	Canella et al. [61], Welker et al. [40]
Peglion et al. [56]	Tg(Huc:GFP)	U87	mKate2	20–50	72 hpf	Orthotopic; midbrain	32 °C	
Wilms et al. [17]	Wild type, Tg(olig2:GPF), Tg(βact:Grx2)	U343-MGA	Fluorescently labelled	300	3.5 hpf	Orthotopic; blastula	33 °C	
Xu et al. [59]	Not mentioned	U251-HF	GFP	50	36 hpf	Orthotopic; forebrain, midbrain	32 °C	Canella et al. [61], Welker et al. [40]
Zhang et al. [33]	Not mentioned	U251/U87:HMC3 (2:1)	LV3, LV10	200 (5 nL)	3.5–4.5 hpf	Heterotopic; yolk sac (centre)	28 °C (1 h), 31 °C	Geiger et al. [44] (modified)
Benson et al. [60]	Not mentioned	U87	nlsCrimson	50	48 hpf	Heterotopic; yolk sac (edge)	Not mentioned	Yang (2013), Vittori et al. [29], Vargas-Patron et al. [19]
Caja et al. [43]	Tg(fli1:EGFP)	Patient-derived	Fluorescently labelled	400	48 hpf	Heterotopic; duct of Cuvier	33 °C	Ren (2017)
Porčnik et al. [25]	Wild type; AB	U87, GSCs (NCH421K), U373	DsRed, GFP	50–100 (5 nL)	52 hpf	Orthotopic; brain	31 °C	Porčnik et al. [25]
Rudzinska-Radecka et al. [42]	Tg(fli1:EGFP)	U87	CM-DiI	500 (5 nL)	6 hpf, 48 hpf	Orthotopic; hindbrain ventricle	35 °C	Wehmas et al. [32], Marques (2009), Berens (2016)
Wang et al. [26]	Wild type; AB	U251, U87	Fluorescently labelled	300	48 hpf	Heterotopic; yolk sac	35 °C	Vargas-Patron et al. [19]
Wu et al. [30]	Wild type; AB/Tübingen (AB/TU)	U87-MG	DiO	100	48 hpf	Orthotopic; brain	32 °C	Yu et al. [18]
Zhang (2021)	Wild type; AB	U87-MG	RFP	50–100 (5 nL)	48 hpf	Orthotopic; brain	28 °C	
Zhong et al. [27]	Not mentioned	U251	CM-DiI	2 × 10^4^cells/μL	48 hpf	Heterotopic; yolk sac	32 °C	
Angom et al. [36]	Casper (roy;nacre)	Patient-derived (GBM1A, GBM22)	GFP, luciferase	50–100, 200–300	36 hpf	Orthotopic; brain ventricle	32 °C	
Cam et al. [37]	Casper (roy;nacre)	DBTRG, SJ-GBM2	GFP (LV)	25–50	36 hpf	Orthotopic; midbrain-hindbrain boundary	32 °C	Welker et al. [40]
Nešović et al. [31]	Tg(fli1:EGFP) × Casper	Patient-derived (GBM22), D54-MG	CM-DiI, td-Tomato	25–50 (1 μL)	72 hpf	Orthotopic; midbrain-hindbrain boundary	32 °C	
Umans et al. [45]	Tg(fli1:EGFP) × Casper	patient-derived (GBM22), D54-MG	CM-DiI, td-Tomato	25–50 (1 μL)	72 hpf	Orthotopic; midbrain-hindbrain boundary	32 °C	
Yu et al. [18]	Wild type	U251	CM-DiI	200 (4.6 nL)	48 hpf	Heterotopic; yolk sac	28 °C	
Vargas-Patron et al. [19]	Wild type	ATCC® CRL-1718™ human astrocytoma	CellTrace™ Far Red	100 (1–3 nL, 3000 cells/mL)	48 hpf	Heterotopic; yolk sac	33 °C	
Banasavadi-Siddegowda et al. [38]	Casper (roy;nacre)	Patient-derived (GBM neurospheres)	GFP	50	36 hpf	Orthotopic; midbrain-hindbrain boundary	32 °C (5d), 28 °C	
Fan et al. [20]	Wild type	U251, U87-MG	CM-DiI	200 (10 μL)	48 hpf	Heterotopic; yolk sac	28 °C (1 h), 32 °C	
Gamble et al. [47]	Tg(fli1:EGFP)	U251-MG	Fluorescently labelled	25–100	48 hpf	Orthotopic; hindbrain ventricle	33 °C	
Pudelko (2018)	Wild type; Tupfel long fin (TL), Tg(fli1:EGFP), Tg(mpeg1:mCherry), Tg(gfap:GFP), Tg(Huc:GFP), Zebrabow	U343-MGA, patient-derived (#18,#3101, #3024)	GFP, CM-DiI	100	3.5 hpf	Orthotopic; blastula	33 °C	
Breznik et al. [28]	Wild type; AB	U373/U87:MSCs (1:1)	dsRED, GFP, CM-DiI, DiO	50–100 (5 nL)	52 hpf	Orthotopic; brain	31 °C	
Canella et al. [61]	Not mentioned	U251-HF	GFP	25–50	36 hpf	Orthotopic; midbrain-hindbrain boundary	32 °C	Welker et al. [40]
Lai et al. [54]	Tg(kdrl:mCherry)	U373	GFP	300–400	48 hpf	heterotopic; yolk sac	31 °C (1 h), 35 °C	
Schnekenburger et al. [21]	Wild type	U373, HS683 oligodendroglial	CM-DiI	100–200	48 hpf	Heterotopic; yolk sac	28.5 °C	Florean (2016) (modified)
Vittori et al. [29]	Wild type; AB	U87	DsRed	50–100 (3 × 10^7^ cells/mL)	52 hpf	Heterotopic/orthotopic; yolk sac, brain	31 °C	Welker et al. [40]
Welker et al. [41]	ABLF (ABxTupfel long fin) × Casper (roy;nacre)	Patient-derived (GBM9 neurospheres)	GPF	50–75	36 hpf	Orthotopic; midbrain-hindbrain boundary	32 °C	
Zeng et al. [46]	Tg(fli1:EGFP) × Casper	U251, U87	RFP, GFP	200–500 (10^7^cells/mL)	72 hpf	Orthotopic; brain	33 °C	
Hamilton et al. [55]	Tg(mpeg1:mCherry), irf8^−/−^ mutant	U251, U87	CM-DiI	8–30	72 hpf	Orthotopic; midbrain (optic tectum)	34 °C	
Ren et al. [51]	Tg(fli1:EGFP)	U87	RFP	10^4^	48 hpf	Heterotopic; yolk sac	35 °C	
Wehmas et al. [32]	Wild type; tropical 5D (T5D)	U87-MG	CM-DiI	50–100	42–72 hpf	Orthotopic; hindbrain ventricle	33 °C (± 1 °C)	
Welker et al. [40]	ABLF (ABxTupfel long fin) × Casper (roy;nacre)	Patient-derived (GBM9 neurospheres, X12)	GPF	50, 100	36 hpf	Orthotopic; midbrain-hindbrain boundary	32 °C	
Yang et al. [50]	Tg(fli1:EGFP)	U87-MG	DiO	9.2 nL (25 × 10^7^ cells/mL)	48 hpf	Orthotopic; brain ventricle	28 °C	
Rampazzo et al. [57]	Tg(hsp70:dkk-GFP)	Patient-derived	Luciferase	100–150 (20-50nL)	168 hpf	Orthotopic; midbrain-hindbrain boundary	34 °C	
Yang et al. [48]	Tg(fli1:EGFP)	U87-MG	RFP	300	48 hpf	Heterotopic; yolk sac (centre)	35 °C	
Yang et al. [49]	Tg(fli1:EGFP)	U87-MG	RFP	200	48 hpf	Heterotopic; yolk sac	28 °C (1 h), 31 °C, 33 °C or 35 °C	Nicoli (2007)
Li et al. [53]	Tg(VEGFR2:G-RCFP)	U87	DsRed	(10-30nL, 10^8^cells/mL)	48 hpf	Heterotopic; perivitelline space (PVS)	28 °C	Nicoli (2007)
Zhao et al. [23]	Wild type; Tupfel long fin (TL)	U87-MG, U87-L	Luciferase	6 hpf	6 hpf	Heterotopic; yolk sac (centre)	28 °C	
Geiger et al. [44]	Tg(fli1:EGFP)	U251	RFP	3.5–4.5 hpf	3.5–4.5 hpf	Heterotopic; yolk sac	28 °C (1 h), 31 °C	

### Data items

The following data were extracted from each study: title, first author, year of publication, zebrafish strain, cancer cell line, cell labeling technique, injected cell number, suspension volume and concentration, time and site of injection, xenograft maintenance temperature, study aim, and respective zebrafish model evaluation by the researchers.

### Synthesis methods

Comparison of the reviewed studies designated similarities and the studies were grouped accordingly. Developmental stage at the time of the xenotransplantation (i.e., embryo or larvae) and injection site (i.e., yolk sac, brain, perivitelline space, etc.) among the extracted data items were the main classification parameters.

## Results

### Zebrafish strain

Research teams have picked different zebrafish wild-type stains as well as transgenic zebrafish strains with DNA fragments embedded in their genome. From the papers reviewed herein, 16 [17–32] research teams have raised wild-type zebrafish until the desired developmental stage before GBM cells xenotransplantation. Among the wild-type strains Tupfel long fin (TL) (*n* = 2) [22, 23], AB (*n* = 6) [24–26, 28, 29, 33], AB/Tübingen (AB/TU) (*n* = 1) [30], Tübingen (TU) (*n* = 1) [31], and tropical 5D (T5D) (*n* = 1) [32] have been used. Transgenic zebrafish have become a powerful tool for modern laboratories as they can be used for various experimental applications, including generating transparent mutants and achieving stable overexpression of fluorescent proteins in cells of interest. To prevent pigment formation commencing at 24 hpf (hours post-fertilization) zebrafish can be treated with 1-phenyl-2-thiourea (PTU) that demonstrates inhibitory effect on formation of melanophores. To avoid this extra step in the protocol, that also lies risk of toxicity and teratogenicity, researchers have used transparent mutants to ensure optical transparency. Casper mutant strains (roy;nacre double mutants) have been used in 5 of the reviewed research papers [34–38], while the mitfa^b692/b692^; ednrba^b140/b140^ transgenic strain used by Gabler et al. [39] could also render the fish devoid of pigmented melanocytes. A strategy of crossing ABLF (ABxTupfel long fin) wild strains with Casper mutants was followed in 2 papers [40, 41]. Tg(fli1:EGFP) strain facilitates the investigation of blood vessels development, outlining the endogenous vasculature with enhanced green fluorescent protein (EGFP) and has been broadly utilized for GBM zebrafish xenotransplantation models (n = 11) to study angiogenesis and metastasis [22, 42–51]. Two of these research teams combined the optically translucent vascular reporter line with a Casper mutant strain to additionally avoid pigmentation [45, 46]. Apart from the fli1 promoter, kdrl, the zebrafish homolog of the VEGF2 receptor has been utilized in vascular-specific zebrafish to drive expression of EGFP (*n* = 1) [52], green reef coral fluorescent protein (GRCFP) (*n* = 1) [53], or the red fluorescent protein mCherry (*n* = 2) [34, 54] to mark the vascular endothelial cells. The use of Tg (mpeg1:mCherry) [22] and Tg (mpeg1:EGFP) [55] transgenic zebrafish strains has allowed for visualization and tracking of macrophages, including microglia, while co-employing irf8^−/−^ mutants that lack microglia can provide a better picture of the role of microglia in GBM cell growth and survival [55]. Endogenous zebrafish neural stem cells/astrocytes can be marked in Tg (gfap:GFP) strains [22] and Tg (olig2:GPF) constitutes an oligodendrocyte transgenic line [17]. With Tg (Huc:GFP) [22, 56] GFP expression is restricted to the neurons. The amazing potential of the zebrafish for transgenic manipulation allows for development of strains tailored to the particular research objective. For example, Tg (βact:Grx2) strain overexpresses the oxidoreductase glutaredoxin 2 [17], in Tg (hsp70:dkk-GFP) strain Wnt signaling can be conditionally suppressed by overexpression of DKK1 [57] and Zebrabow strain can be used to acquire in vivo multicoloring images [22]. The zebrafish strain employed for the xenotransplantation models was not mentioned and could not be inferred in 6 of the reviewed papers [27, 33, 58–61].

### Cell line and labeling

The cancer cell lines injected vary and can be either laboratory-derived or patient-derived xenografts (PDX). Among the laboratory cell lines, the malignant glioma cell lines U87 (*n* = 20) [20, 23, 25, 26, 29–33, 42, 46, 48–53, 55, 56, 60] and U251 (*n* = 12) [18, 20, 26, 27, 44, 46, 47, 52, 55, 58, 59, 61] predominate. When Ai et al. [52] testified the ability of the zebrafish model to reveal GBM intertumor heterogeneity and intratumor homogeneity, they found that models injected with U87, U251, G1261, C6 cell lines, or patient-derived cells were able to recapitulate the distinct histological features of each tumor. U87 cells comprise a highly vascularized tumor with limited invasion ability into the surrounding parenchyma and are widely used in studies of GBM angiogenesis, while U251 cells display extensive growth pattern [46, 52].

Primary patient-derived GBM cells have been widely used, displaying tumor initiating potential in zebrafish embryos and thus successfully establishing PDX models (*n* = 12) [22, 34, 36, 38, 40, 41, 43, 45, 46, 52, 57, 58]. As fresh surgically resected material failed to proliferate, when transplanted into zebrafish, different culture methods were employed, including organoid, neurosphere, and attached culture, with the last displaying the highest success rate [52]. GBM9 neurospheres [40, 41] and primary patient-derived neurospheres (GBMNS) [38] have also been used by research teams. The xenografted patient-derived GBM cells infiltrative growth in the zebrafish was patient-dependent ranging from highly infiltrative to demarcated, phenocopying the patient MRI [34, 52]. Caja et al. [43] used patient-derived mesenchymal cultures (U3031 and U3034 MG/MS) from grade IV GBM biopsies, and Umans et al. [45] developed a PDX model utilizing the GBM22 PDX line.

Glioma stem cells (GSCs) driving the progression of GBM have also been successfully transplanted into zebrafish embryos. For this purpose, different research teams injected GBM3 [24], GSC23 [35], or NCH421K cells [25] into zebrafish embryos. Another strategy that has been employed for the zebrafish xenograft models concerned mixing GBM cells (U251 or U87) with HMC3 microglia cells in 2:1 ratio [33] or GBM cells (U373 or U87) with MSCs in 1:1 ratio. Other cell lines that have been utilized in GBM zebrafish xenograft model research include U343 (*n* = 2) [17, 22], U373 (*n* = 3) [21, 25, 54], BTL1528 and FGFR4-KD [39], the ATCC® CRL-1718™ human astrocytoma cell line [19], D54-MG [45], DBTRG and SJ-GBM2 [37], and the HS683 oligodendroglial cell line [21].

Labeling the injected GBM cells is crucial to follow their proliferation and infiltration path inside the xenograft zebrafish model. The cell labeling techniques include the luciferase enzyme reaction with the luciferin substrate [23, 34, 36, 57] and fluorescent dyes, such as the DsRed [25, 28, 29, 53], the lipophilic fluorescent red dye CM-DiI [18, 20–22, 27, 28, 31, 32, 42, 45, 55], DiO labeling [28, 30, 50], and the CFSE fluorescent dye [52]. Cell labeling can also be achieved by expression of optical reporter genes and fluorescent proteins, such as the RFP [33, 44, 46, 48, 49, 51] the GFP gene [22, 25, 28, 34, 36–41, 46, 54, 59, 61], the Zs Green fluorescent protein [24], mCherry red fluorescent protein [45], td-Tomato fluorescent protein [45], the E2 Crimson fluorescent protein [60], the CellTracker™ Green CMFDA fluorescent protein [35], or the CellTrace™ Far Red fluorochrome [19].

### Number of cells

Widely varied number of GBM tumor cells have been xenotransplanted in zebrafish embryo models ranging from 8 to 10^4^ in the papers reviewed herein. Most research teams tend to inject a number of cells between 50 and 200 (50–75 (*n* = 1)[41], 50–100 (*n* = 6)[25, 28, 29, 32, 33, 36], 50–200 (*n* = 1)[44], 100 (*n* = 6) [19, 22, 23, 30, 40, 52], 125 (*n* = 1) [31], 150 (*n* = 1) [34], 150–200 (*n* = 1) [24], 100–150 (*n* = 1) [57], 100–200 (*n* = 1) [21], 200 (*n* = 4) [18, 20, 33, 49])), with 50–100 and 100 cells injections predominating. Injecting more than 200 GBM cells (200–10^4^ cells) was adopted by 10 teams (200–300 (*n* = 1) [36], 250 (*n* = 1) [35], 200–500 (*n* = 1) [46], 300 (*n* = 3) [17, 26, 48], 400 (*n* = 1) [43], 450 (*n* = 1) [54], 500 (*n* = 1) [42], 10,000 (*n* = 1) [51]), while injecting less than 50 cells seems to be the least common practice for the zebrafish GBM model development (8–30 (*n* = 1) [55], 20–50 (*n* = 1) [56], 25–50 (*n* = 3) [37, 45, 61]). Umans et al. [45] observed that when they transplanted more than 50 cells into the zebrafish brain at 3 dpf (days post-fertilization), the cells were trapped within the ventricular space, leading to necrosis. They also noted that slight changes in the applied pressure could result to changes in the number of cells implanted. Zeng et al. [46] detected an initial tumor volume reduction associated with the cell line, which was observed with U87 cells and not with U251 cells. The GBM cell line seems to play a crucial role in the required number of cells for successful xenotransplantation. Welker et al. [40] recorded the dose-dependent effect of GBM cell injections on the zebrafish lethality for two patient-derived cell lines, serum-grown X12 and GBM9 neurospheres and noted a median zebrafish survival of 5dpt for injection of 51–90 GBM9 neurospheres and 10dpt for injection of 51–90 GBM9 cells, highlighting the role of the cell line for the cell number injection optimization. Yang et al. [49] described the cell number-related angiogenesis as hardly detectable, slight, or highly observable for injection of 20, 50, 100–200 cells, respectively, with injection of 200 cells yielding the most significant neovascularization along with an acceptable zebrafish survival rate (82%). They also estimated the survival rates for different injection sites and the results revealed the ability of the yolk sac to sustain higher number of cells more robustly. Finally, in general, it was observed that the GBM cell suspensions were highly dense (10^7^–10^8^ cells/mL) and the infusion volumes were small (mostly in the nL order of magnitude).

### Injection time

Various time points throughout the zebrafish development have been tested for their ease to be integrated into the experimental procedure and for the zebrafish capacity to host the injected GBM cells. Early embryonic to later embryonic and larval zebrafish GBM models have been developed. Xenografts in zebrafish are usually performed during embryonic stages, since the adaptive immune system has not developed yet and, thus, immunosuppression is not required. The embryonic stages classification mentioned below is based on Kimmel et al. [62].

The blastula period refers to the zebrafish developmental stage from 2^1^/_4_ h to 4^2^/_3_ h post-fertilization, including 128-cell, 255-cell, 512-cell, 1 k-cell, high, oblong, sphere, dome, and 30%-epiboly stages. For embryonic models, injection at 3.5 hpf, marked as the 1 k-cell stage (*n* = 2) [17, 22], or at 3.5–4.5 hpf, marked as the oblong to sphere stage (*n* = 2) [33, 44], facilitated lining up of hundreds of embryos in agarose molds and granted rapid transplantation, while reducing the need for precise orientation and sedation. Also, it has been claimed that early blastula embryos send homing signals to the GBM cells and support them trophically [22]. GBM cells injection at the gastrula period (5^1^/_4_ to 10 h post-fertilization), specifically at 6 hpf, marked as the shield stage (when the embryonic shield becomes visible from the animal pole), has also been employed (*n* = 1) for an embryonic GBM zebrafish model [23]. This time point has also been utilized for embryotoxicity evaluation of ITCs (isothiocyanates) before xenograft larval model development [42].

Injection during the pharyngula period (24–48 hpf), at 24 hpf (prim-5) (n = 2) [34, 35] when the embryo has developed to the phylotypic stage, or at 36 hpf (prim-25) (n = 7) [36–38, 40, 41, 59, 61] has also been successful, allowing for GBM model development. The most commonly used time point for GBM cells microinjection in zebrafish (n = 21), however, is 48 hpf, marked as the end of the pharyngula period and the start of the hatching period (48–72 hpf) [18–21, 24, 26, 27, 30, 31, 33, 39, 42, 43, 47–51, 53, 54, 60]. This time point renders an adequate developmental stage for the xenograft transplantation experiments. Injection in the course of the hatching period (*n* = 4) [25, 28, 29, 32], between 2 and 3 dpf, has displayed minimal mortality due to injection, while morphogenesis of the primary organ systems has been completed, including a rudimentary, blood–brain barrier that develops fully by 3dpf [32].

It has arbitrarily prevailed to call the zebrafish “embryos” until 3 dpf (72 hpf) and then “larvae” regardless of their hatching state. Zebrafish have been raised until the early larval stage, before xenotransplantation at 72 hpf (*n* = 6) [45, 46, 52, 55, 56, 58]. At 72 hpf the blood–brain barrier existence and functionality has been proved [46] and CNS angiogenesis has been sufficient [45]. Ai et al. [52] have compared the success rate and growth of implanted GBM xenografts for orthotopic microinjections from 2 to 5 dpf, and considered 3 dpf as the optimal injection time point, enabling a long observation time window (10 days) before lethality became significant. 3 dpf larvae are still fragile, though, and require careful handling, while the short time window marked by sharp decrease in GBM cell invasion at 96 hpf – attributed to zebrafish immune response, altering the tumor microenvironment – sets another limitation [56]. Finally, a larval GBM xenograft model (*n* = 1) has been developed at 7 dpf (168 hpf), allowing for the investigation of the Wnt pathway effect on patient-derived GBM cells [57].

### Injection site

**Fig. 2:**
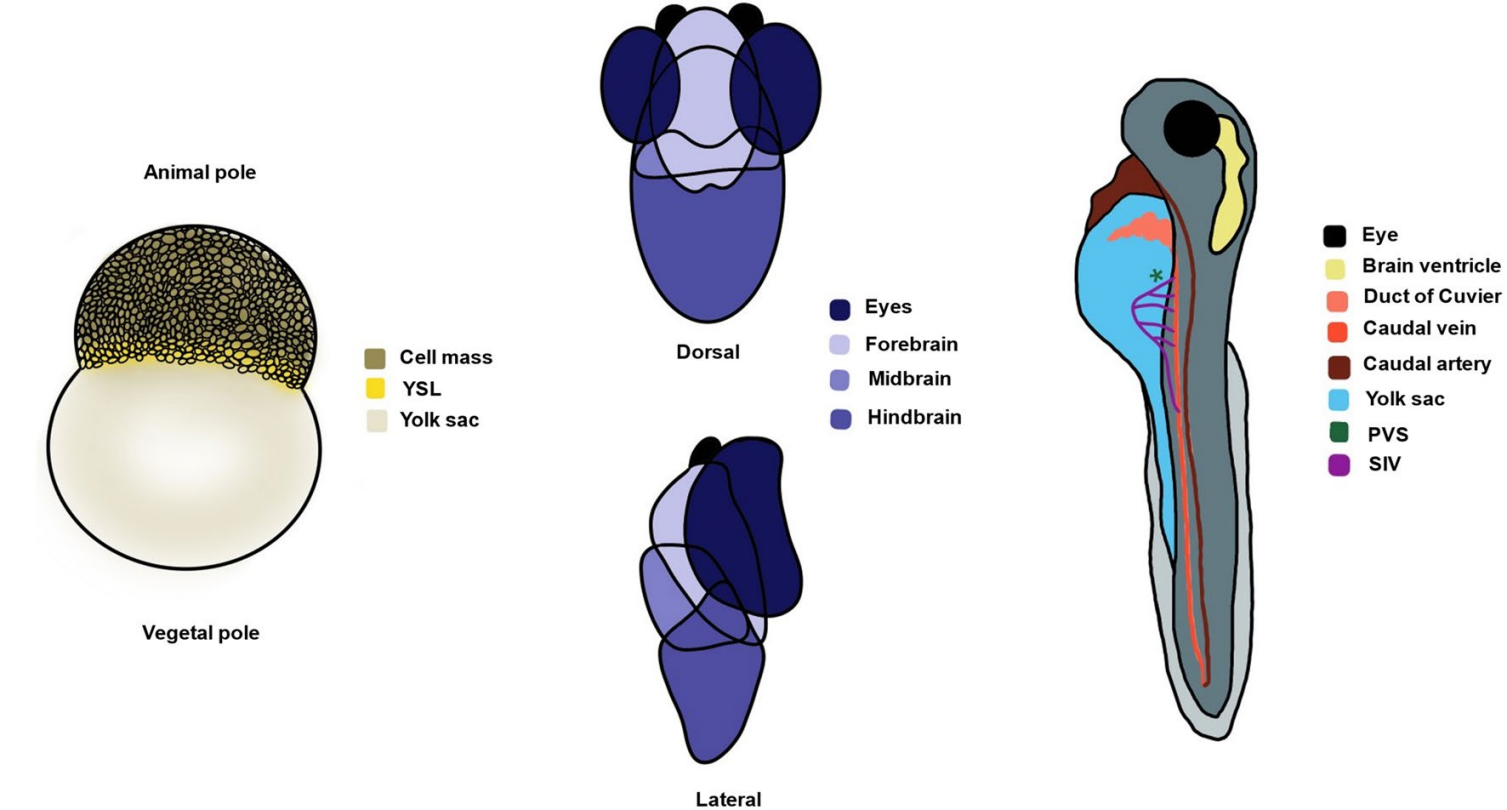
Zebrafish embryo anatomy and possible injection sites for GBM xenografting. Left: blastula period blastodisc allowing for injection into the cell mass or the yolk sac for the establishment of early embryonic xenograft models. Middle: brain regions of the developing zebrafish constituting possible injection sites for the establishment of late embryonic/larval orthotopic xenograft models. Right: alternative injection sites for orthotopic (brain ventricle) or heterotopic (duct of Cuvier, yolk sac, PVS) xenotransplantation. *YSL* yolk syncytial layer, *PVS* perivitelline space, *SIV* subintestinal vessels

Various locations of the developing zebrafish have been utilized as injection sites for GBM cells at the embryonic and larval stages (Fig. 2). From the early embryonic zebrafish models reviewed herein (*n* = 5), 2 involved injection into the blastoderm [17, 22] and 3 into the yolk sac [23, 33, 44]. GBM cells injected into the blastula migrated to the CNS of the developing zebrafish embryo, leading to the development of orthotopic intracranial tumor masses by 24hpi. GBM cells migratory behavior was independent of the transplantation site—apically or basically—within the blastoderm and the majority of them traveled to the forebrain/midbrain [22]. The transplantation procedure into the blastodisc was automatable, did not require sedation, and was, thus, considered robust. Microinjection into the embryonic yolk sac compared to the cell mass halved the zebrafish mortality rate, providing a xenograft model less vulnerable to tissue microenvironment signaling, which could otherwise lead to cancer cells phenotypic alterations [33, 44]. The yolk covers the nutritional needs of the xenograft model, not calling for supplemental feeding up until 7 dpf, and human GBM cells were reported to survive within the zebrafish host no less than this time point. Zebrafish endothelium, while at first developing separately from the yolk sac, then extended and directly contacted the GBM mass, allowing for studying of GBM angiogenesis stimulation capacity [44]. The yolk sac embryonic zebrafish model has also validated bioluminescence imaging as scanning method for antitumorigenesis compound screening in zebrafish embryos [23].

Among the reviewed late embryonic and larval zebrafish GBM xenograft models (*n* = 41), 24 aimed for orthotopic [12, 24, 25, 28, 30, 32–34, 36–38, 40–42, 45–47, 50, 52, 55–57, 59, 61] and 17 for heterotopic [18–21, 26, 27, 31, 35, 39, 43, 48, 49, 51, 53, 54, 58, 60] transplantation. The zebrafish embryos’ brains mimic well the human GBM microenvironment with the presence of neuronal tracts and laminin at the early stages, rendering the brain parenchyma as a suitable in vivo physiological matrix to study tumor growth [25, 28, 29]. 7 research teams picked the midbrain–hindbrain boundary as injection site for their orthotopic zebrafish model [37, 38, 40, 41, 45, 57, 61]. This site has been favored as an endogenous Wnt-rich site to investigate the effect of the Wnt pathway in GBM [57] and was established as a landmark for cell transplants to ensure consistency [40]. Recruited fish with clearly visible midbrain–hindbrain boundary developed detectable gliomas at 5dpt that had robustly grown by 10dpt [61] and survived for 20dpt [38], also extending finger-like processes, and migrating along the vascular network [45]. 4 of the reviewed xenotransplantation models injected GBM cells into the larval midbrain [34, 52, 55, 56], 2 of which into the optic tectum (TeO) [52, 55]. Zebrafish midbrain injection showed 93% success rate [34] and was reported as substantially recapitulating human GBM pathophysiology and able to mimic the pattern of GBM cell invasion in human vessels [56]. The optic tectum of the midbrain has been considered optimal for orthotopic xenografting, exhibiting high success rate and permitting 10-day observation time window [52], while also stimulating intensive microglia response and recruitment to the transplantation site [55]. Hindbrain [24] and forebrain [59] injection have been employed for GBM xenografting to a limited extent. Zebrafish brain ventricles were the injection site of choice in 5 of the reviewed articles [32, 36, 42, 47, 50]. The significance of injection site location and microenvironment for realistic GBM behavior was exemplified by injection into the hindbrain ventricle by 3 research teams [32, 42, 47].

Heterotopic transplantation into the larval yolk sac (*n* = 14) [18–21, 26, 27, 31, 39, 48, 49, 51, 54, 58, 60], perivitelline space (PVS) (*n* = 2) [35, 53] or the duct of Cuvier (*n* = 1) [43] has also been employed. GBM cell injection into the yolk sac of zebrafish larvae was followed by no increase in the fluorescent signal intensity at 1–3dpt in contrast to orthotopic injection [29]. Yolk sac engraftment rate reached 73%, and microtumors became larger and grew eccentrically from 24 to 72hpi, not only becoming detectable but also starting to regress (some perished within 24hpi) and only occasionally invading to distant sites (tail) via circulation [19]. However, yolk sac provides for a spacious matrix to host the GBM xenograft favoring proliferation and facilitating GBM cells phenotype conservation by decreasing susceptibility to tissue microenvironment signaling while constituting a nutrient-rich acellular compartment [19]. Yang et al. [49] systematically established a reliable tumor GBM xenograft zebrafish model for angiogenesis evaluation with potential use for immunopharmacology studies and anti-angiogenic drug screening. Opting for acceptable survival rate and the highest efficiency, they chose the yolk sac (82% survival rate while tolerating maximum GBM number) over embryonic cell mass (> 80% mortality rate at 48hpi) and SIV (significant reduction in survival rate with injected cell number increase). No significant difference as for positive angiogenic response between the SIV and the yolk sac injection was observed. However, the yolk sac begins to shrink at 3dpi, because nutrition is absorbed by the zebrafish, leading newly formed vessels to lose their morphology and become curved or twisted. The duct of Cuvier, also known as the common cardinal vein, has also served injection into the bloodstream to study GBM invasiveness, extravasation and metastatic potential [43]. Finally, the perivitelline space (PVS) near the subintestinal vessels (SIV) has been used to investigate GBM angiogenesis mechanisms [53] and evaluate GBM aggressiveness [35].

### Temperature

While constituting an advantageous, promising animal model, zebrafish display an inherent maintenance temperature limitation compared to mouse xenograft models, given the optimal temperature differential between zebrafish embryos and human cells. Human glioma cells typically develop at 37 °C, reflecting the human body temperature that delivers the mammalian cells metabolic requirements, while wild zebrafish reside in cooler tropical natural habitats, below 30 °C. Therefore, it is imperative that a compromise is made between the optimal temperature for the fish (28.5 °C) and the xenografted GBM cell lines. Reportedly different post-injection incubation temperatures have been applied in the literature ranging from 28 °C to 35 °C. Intermediate incubation conditions at 32 °C (*n* = 11) [24, 31, 33, 36, 37, 40, 41, 45, 56, 59, 61], 33 °C (*n* = 10) [15, 17, 19, 22, 26, 32, 34, 43, 46, 52], or 31 (*n* = 3) [25, 28, 29] were the most frequently used. Gradual acclimatization of xenografted fish to develop 32 °C increased the survival compared to instant alteration from 28 °C to 32 °C [45, 56]. Incubation at 33 °C did not affect the BBB of the zebrafish embryos [26], as well as the embryo viability, while the GBM cells adequately retained their migrating and proliferating potential [19, 32] following a steady-state growth at a lower rate compared to 37 °C [46]. Although in some cases, temperature rise above 32 °C came with lethal developmental changes for the zebrafish embryos [56], researchers have maintained their xenografted zebrafish at temperatures as high as 34 °C (*n* = 4) [35, 39, 55, 57] or 35 °C (*n* = 4) [30, 42, 48, 51] to accommodate a more desirable temperature for glioma cell growth. Other researchers followed a different approach, incubating the xenotransplanted zebrafish at 28 °C (*n* = 4) [30, 42, 48, 51] or 28.5 °C (*n* = 1) [21], typical for zebrafish maintenance, but suboptimal for the GBM cells. In some of the research papers (*n* = 3) reviewed herein, the incubation temperature conditions were monitored to favor the zebrafish embryo development right after the xenotransplantation (28 °C for 1 h) and then raised to accommodate the GBM cells proliferation (31 °C (*n* = 2) [33, 44], 32 °C (*n* = 1) [20], 31 °C, 33 °C, and 35 °C (*n* = 1) [49]), or the opposite, maintaining the xenografted zebrafish at 32 °C for 5 days before lowering the incubation temperature at 28 °C [38]. Yang et al. [49] employed incubation at 28 °C for 1 h, before maintenance at 31, 33, or 35 °C to determine the optimal temperature for glioma cell-induced angiogenesis. Temperature elevation above 35 °C resulted in necrotic tissue and twisted body phenotypes with high embryo mortality rate. Angiogenesis measured by number and length of newly formed vessels was significantly more prevalent at 35 °C, implying that that the higher temperature results in higher cellular viability of injected tumor cells. Geiger et al. assessed the proliferation, colony formation ability, and radiosensitivity of U251-RFP cells and found them similar at any temperature above 28 °C [44]. One last incubation approach involved keeping the xenografted fish at 31 °C for 1 h before incubation at 35 °C for the rest of the assay [54]. The maintenance temperature of the xenotransplanted zebrafish embryos was not mentioned and could not be inferred in 2 of the reviewed papers [58, 60].

## Discussion

Glioblastoma is the most common primary brain tumor in adults. The poor prognosis, the chemoresistance, and the treatment challenges posed by its highly infiltrative nature, genetic heterogeneity, and protection by the blood–brain barrier make it imperative to find innovative and effective treatment approaches [30]. The challenge has stimulated the interest of the scientific community, opting for novel animal models for preclinical studies, with the zebrafish comprising a promising alternative to traditional murine models. Zebrafish embryos possess numerous advantages as they develop rapidly, are optically transparent, and share high genetic homology with humans, allowing for translatable brain cancer research and high-throughput drug screening [15]. This highly attractive – though relatively new – model has been increasingly used for GBM xenografting studies by different research teams without a standardized methodology. The GBM cell line and number, the zebrafish strain, the injection site, and the time point of the injection as well as the post-transplantation maintenance temperature make protocol parameters requiring optimization.

There are many different GBM cell lines available, each with a unique set of characteristics (i.e., growth rate, invasiveness, response to treatment). U87 and U251 comprise particularly popular choices, as they are well-established cell lines. U87 cells have been frequently used for studying GBM angiogenesis, while U251 cells display an extensive growth pattern and thus allow for investigation of GBM proliferation and invasion. Patient-derived xenograft zebrafish models are emerging as a promising tool, confirming the clinical relevance of this animal model faithfully recapitulating GBM behavior in vivo [5]. The ability to assess the aggressiveness of the original patient tumor and make predictions for its invasion and metastatic potential renders zebrafish as a valuable tool for prognosis. Compared to traditional murine models, zebrafish embryos–larvae require a minimal number of GBM cells allowing for the generation of more xenografts from a single patient [15]. Labeling the xenografted GBM cells is substantial to follow their proliferation and infiltration path. For this purpose, various approaches have been employed including the luciferase enzyme reaction, fluorescent dyes (DsRed, CM-DiI, DiO), and the optical reporter genes RFP and GFP. The GBM cell line seems to play a crucial role in the required number of cells for successful xenotransplantation and correlations have also been observed between the injected cell number and the zebrafish survival rates as well as the angiogenesis. Α number of cells between 50 and 200 are usually injected, with 50–100 and 100 cells injections predominating. A period of optimization before the establishment of the xenograft model is required to test if the cell line has toxic effects for the embryo–larva or higher/lower injection densities are required. Apart from the widely used wild-type zebrafish strains, transgenic strains have been developed, such as the Casper strain, producing reduced pigmentation and aiding in imaging studies. Other transgenic strains have been engineered to stably overexpress fluorescent proteins in targeted cells, allowing researchers to visualize and track them in vivo.

The developmental stage of the zebrafish at the time of the transplantation as well as the injection site are critical parameters for the success of the xenografting protocols. Zebrafish embryos develop rapidly, undergoing significant changes in their physiology, anatomy, and immune system. Xenografts in embryonic stages outperform larval xenografts as they do not require immunosuppression since the adaptive immune system has not fully developed yet. Injection at the blastula stage, at 3.5–4.5 hpf, grants rapid transplantation, does not require sedation and displays reduced need for precise orientation. The most commonly used time point for GBM cells microinjection in zebrafish, though, is the end of the pharyngula period–start of the hatching period, at 48 hpf. Zebrafish at 48 hpf possess a more advanced developing brain compared to 24 hpf with more clearly defined regions for xenotransplantation as well as circulatory and lymphatic systems favoring the tumor development and metastasis and allowing for GBM study in a more physiologically relevant microenvironment. As the zebrafish body plan is formed at 2 dpf, xenotransplantation at this time point reduces the likelihood of passive transport during gastrulation [63]. In addition, xenotransplantation at 48 hpf provides a longer time window (more than 7 dpf) for observations [64] while also holding relatively less ethical concerns compared to earlier or later stages. At 72 hpf the blood–brain barrier is fully functional and CNS angiogenesis has been rendered sufficient.

Injection into the blastoderm or the yolk sac of the blastula stage is automatable and can ultimately lead to the development of orthotopic intracranial tumor masses. Microinjection into the embryonic yolk sac supports the GBM cells trophically up until 7 dpf and has been observed to double the zebrafish survival compared to microinjection into the cell mass. Orthotopic predominated compared to heterotopic GBM xenotransplantation in late embryonic and larval zebrafish models as the zebrafish brain highly resembles the human brain microenvironment. Injection into the midbrain–hindbrain boundary has been the most widely tested injection site favoring successful xenotransplantation. Among the heterotopic injection sites, the yolk sac provides for a spacious and nutrient-rich matrix to host the GBM xenograft facilitating GBM cell proliferation and phenotype conservation. Injection into the yolk sac has been successful in angiogenesis evaluation and immunopharmacology studies. However, it begins to shrink at 3dpi because nutrition is absorbed by the zebrafish. The perivitelline space and the duct of Cuvier serve as less commonly chosen alternative injection sites. Additionally, the injection technique should be optimized to minimize the potential harm of the zebrafish embryo and ensure the accuracy and reproducibility of the injection.

Finally, temperature is an important factor to adjust when performing a zebrafish xenograft model for glioblastoma considering the difference of the optimal temperature between the zebrafish and the GBM cells, resulting in the employment of suboptimal temperatures for both species. The post-injection incubation temperatures in the literature range from 28 °C to 35 °C, with intermediate incubation conditions between 31 and 33 °C predominating. Hypothermia reduces cell proliferation and migration of GBM cells in a dose-dependent way, and can even be cytostatic, arresting the cell cycle, reducing the metabolic activity and cytokine synthesis of GBM cells, as well as altering their morphology [65, 66]. Gradual acclimatization of the xenografted zebrafish or development of heat-tolerant transgenic zebrafish able to maintain xenografts at 37 °C could enhance the GBM growth [32].

## Conclusion

Zebrafish xenograft models hold great promise as a tool for preclinical studies as well as in clinical practice when it comes to patient-derived xenografts. This review focuses mainly on zebrafish models for glioblastoma. However, there are many similarities that can be exploited by other cancer models in zebrafish. Ultimately, the ideal zebrafish model for glioblastoma should be generated from a highly automated and accessible process to be utilized in large-scale anticancer drug trials. Our review designated that AB wild-type zebrafish, Casper transparent mutants, transgenic Tg(fli1:EGFP) or crossbreeding of the above-mentioned strains, orthotopically transplanted at 48 hpf with 50–100 U87 cells to study GBM angiogenesis, U251 cells to study GBM proliferation, or PDX to achieve clinical relevance in high density and low infusion volume (nL) gradually acclimatized to 32–33 °C could comprise a successful, conducive and reproducible zebrafish GBM model. However, it is difficult to draw definitive conclusions about the most effective xenografting approach regarding the cell line and number, the zebrafish strain, the developmental stage, the injection site, and the maintenance temperature. It is important to consider the specific research question when designing the research protocol. We recommend that future research focus on addressing the observed methodological inconsistencies to ensure the accuracy and reproducibility of the protocols and scale up the trials identifying novel treatment strategies for glioblastoma.

## Data Availability

The datasets of this study can be made available by the authors upon reasonable request.
